# Effects of Thermal Treatments on the Physicochemical and Flavor Profiles of Chili Powders and Their Derived Chili Oils

**DOI:** 10.3390/foods14173129

**Published:** 2025-09-06

**Authors:** Chunping Jiang, Lijia Zhang, Linman Yu, Zhengfeng Fang, Bin Hu, Hong Chen, Wenjuan Wu, Yuntao Liu, Zhen Zeng

**Affiliations:** 1 College of Food Science, Sichuan Agricultural University, 46# Xinkang Road, Yaan 625014, China; jcp_0207@163.com (C.J.); 18148177150@163.com (L.Z.); 18783204893@163.com (L.Y.); fangzhengfeng@hotmail.com (Z.F.); hubin2555@sina.com (B.H.); chenhong945@sicau.edu.cn (H.C.); 2 College of Science, Sichuan Agricultural University, 46# Xinkang Road, Yaan 625014, China; 72025@sicau.edu.cn

**Keywords:** chili powder, chili oil, thermal treatment, flavor activation, flavor compounds, Maillard reaction

## Abstract

Current research on chili powder and oil has predominantly focused on cultivar selection and oil temperature, while the impact of thermal pretreatment methods on their quality and flavor profiles remains underexplored. In this study, the flavor profiles of raw untreated, stir-fried, oven-baked, and microwaved chili powders (RC, SC, OC, and MC) and their corresponding chili oils obtained through secondary flavor activation (RCO, SCO, OCO, and MCO) were analyzed using E-nose, GC-IMS, HS-SPME-GC-MS, LC-MS/MS, and sensory evaluation techniques. E-nose and GC-IMS 2D topographic plots revealed that thermal treatment increased the concentration of volatile flavor compounds. HS-SPME-GC-MS further detected 220 and 207 volatile compounds in chili powders and oils, respectively, with 74 and 35 identified as differential volatile compounds. Aldehydes ((E,E)-2,4-heptadienal, benzaldehyde), alcohols (1-nonanol, 2-furanmethanol), Maillard reaction products (ethyl pyrazine, 2,3-dimethylpyrazine, and 2-ethyl-6-methylpyrazine), and methyl acetate were significantly enhanced in SC, OC, and MC and their corresponding chili oils. Among them, OC and OCO showed the greatest increase in differential flavor substances. Additionally, all three treatments enhanced the release of taste-active substances and improved sensory overall acceptability. These findings provide new insights for the food industry in optimizing chili product processing.

## 1. Introduction

Chili pepper (*Capsicum annuum* L.), known for its distinctive color, pungency, and aroma, has become an indispensable condiment and natural colorant in the global food industry [1,2]. As reported by the Food and Agriculture Organization of the United Nations (FAO), China’s chili output amounted to 17,134,480 t in 2023, constituting 44.73% of global production [3]. Among them, Erjingtiao chili is regarded as the cornerstone of Sichuan cuisine, characterized by its moderate pungency, suitability for use in both fresh and dried forms, and rich aromatic profile. It is extensively processed into chili powder and chili oil, which have a wide application in seasonings in Sichuanese cuisine seasonings, fermented bean paste, hot pot, and sausages [4,5,6,7]. However, traditional chili powder was typically produced by directly grinding sun-dried peppers, while chili oil was manufactured through the hot oil immersion of conventionally untreated chili powder. These approaches underutilize the flavor potential of chili peppers, often resulting in final products with pronounced raw chili flavor, oily off-flavors, and insufficient roasted aromas.

Flavor, a core quality attribute of chili products, arises from the synergistic interplay of volatile organic compounds (VOCs, e.g., aldehydes, alcohols, acids, hydrocarbons, and esters) and non-volatile taste-active substances (e.g., capsaicinoids, carotenoids, and polyphenols) [8,9,10]. For example, 3-methylbutanal and octanal served as key flavor compounds, imparting acrid, almond, chocolate, cocoa, and fatty characteristics to Erjingtiao chili oil [5]. Meanwhile, capsaicin, dihydrocapsaicin, and related capsaicinoids contributed to its distinctive pungent taste. Notably, the formation and transformation of these compounds are critically influenced by raw material properties, processing techniques, and storage conditions. Among these factors, thermal processing, as a pivotal step in chili product manufacturing, profoundly shapes flavor complexity through Maillard reactions, lipid oxidation, caramelization, and thermal degradation [11,12,13]. Research has shown that short-chain alcohols in chili peppers decrease due to volatilization and esterification in thermal processing [1]. However, certain aromatic alcohols may increase via altered pathways of lipid oxidation or the transformation of carbonyl compounds under drying conditions [3]. Additionally, compounds such as acids, furans, and aldehydes are generated through carbohydrate degradation, lipid oxidation, and the Maillard reaction between amino acids and reducing sugars [14].

In food industry production, emerging pretreatment techniques (e.g., hot-air drying, blanching, freeze-drying, and intermittent microwave) have demonstrated enhanced flavor precursor release via cellular disruption and enzyme activity modulation [1,15,16,17]. For example, blanching pretreatment can effectively remove moisture, preserve the surface color, alter the cell structure, and reduce the loss of capsaicinoids in peppers [3]. Linoleic acid and linolenic acid promote the formation of 1-octen-3-ol during thermal drying, contributing to floral and fruity aromas [17]. Additionally, variable-temperature drying has been shown to increase the content of acids, furans, and sulfides [1]. Moreover, a recent study on the production of chili oil at different temperatures showed that chili oil made at 180 °C had the most pronounced flavor profile [18]. However, the differential regulatory function of three household-prevalent thermal methods, stir-frying, microwaving, and oven-roasting, on chili flavor remain systematically uncharacterized. Stir-frying rapidly heats food through conduction and convection in a wok, while oven-baking relies primarily on thermal radiation and hot-air convection to cook. In contrast, microwaving employs electromagnetic waves to excite water molecules, generating frictional heat that cooks food from the inside out. Crucially, existing research on chili powder primarily focuses on pepper cultivar selection, whereas studies concerning chili oil predominantly concentrate on single processing stages (such as extraction oil temperature), neglecting the dynamic evolution of chili flavor compounds throughout the continuous processing continuum: from thermal pretreatment (for chili powder preparation) to secondary flavor activation via high-temperature oil (for chili oil production) [1,5].

Departing from traditional preparation methodologies, this study employed three thermal treatments (stir-frying, microwaving, and oven-roasting) for chili powder production. Subsequently, the heat-treated chili powder was used as the raw material for secondary flavor activation via immersion in 180 °C rapeseed oils to produce chili oil. Utilizing multidimensional analytical approaches including electronic nose (E-nose), chromatography-ion mobility spectrometry (GC-IMS), headspace solid-phase microextraction gas chromatography-mass spectrometry (HS-SPME-GC-MS), Liquid chromatography tandem mass spectrometry (LC-MS/MS), along with sensory evaluation, systematically elucidated the impact mechanisms of different thermal treatments on the flavor profiles of both chili powders and their corresponding chili oils. This research provides a technical support for optimizing processing technologies in chili-based product manufacturing.

## 2. Materials and Methods

### 2.1. Materials

Dried Erjingtiao chilis were purchased from Muma Mountain, Chengdu city, Sichuan Province, China (awarded the national geographical indication product protection in 2009). Main reagents: 2-Octanol was purchased from TCI (Shanghai, China). The rapeseed oil was purchased from Jinlongyu Co., Ltd. (Chengdu, China). LC-MS pure grade methanol (MeOH), and acetonitrile (ACN) were purchased from CNW Technologies (Dusseldorf, Germany). Unprocessed dried chili powder and commercial chili oil were purchased from a local supermarket (brand: Cuihong, Chengdu, China). All additional reagents used were of analytical grade or liquid phase pure.

### 2.2. Preparation of Chili Powder and Chili Oil with Different Processing

Before sample preparation, chili peppers were subjected to moisture equilibration in a hot-air drying oven (Yiheng Scientific Instruments Co., Ltd., Shanghai, China) at 40 °C for 3 h. Then, they were cut into 3 cm segments and randomly divided into four groups (50 g each) with different thermal treatments. Three thermal treatments were applied: stir-frying in a wok over an C21-RT21E01 induction cooker for 3 min at 300 W (rated power: 2200 W; Midea Life Appliance Manufacturing Co., Ltd., Foshan, China), baking in a preheated oven at 100 °C for 11 min (rated power: 2700 W; Midea Life Appliance Manufacturing Co., Ltd., China), and microwaving on a plate at 300 W for 3 min (Midea Life Appliance Manufacturing Co., Ltd., China). The processed chili segments were ground and sieved through a 25-mesh screen to obtain chili powders (CPs): stir-fried chili powder (SC), oven-baked chili powder (OC), and microwave-treated chili powder (MC). The untreated raw group served as the control (RC).

The preparation of chili oil was based on previous research and slightly adjusted [5]. Oils prepared from SC, OC, MC, and RC were as follows: 250 g of rapeseed oil was heated to 220 °C, then cooled to 180 °C before gradually adding thermal-treated chili powder while stirring continuously until the temperature dropped below 60 °C. Subsequently, the sample was cooled to room temperature, sealed, and stored for 48 h before collecting the supernatant. This process resulted in chili oils (COs): stir-fried chili oil (SCO), oven-baked chili oil (OCO), microwave-treated chili oil (MCO), and untreated raw group chili oil (RCO). Each experiment was repeated three times.

### 2.3. Color Analysis

The L*, a*, and b* values of various chili powders (RC, SC, OC, MC) and chili oils (RCO, SCO, OCO, MCO) were measured using an XD-200 colorimeter (Shanghai Modern Environmental Engineering Technology Co., Ltd., Shanghai, China) [19]. Each sample was tested three times. The total color difference (Δ*E*) in CPs and COs were determined using the following Equation (1):

(1) $$\Delta E\;=\;\sqrt{({L^{*}\;-\;L_{0})}^{2}\;+\;({a^{*}\;-\;a_{0})}^{2}\;+\;{\left(b^{*}\;-\;b_{0}\right)}^{2}}$$

where *L*_0_, a_0_ and *b*_0_ were the color values of RC and RCO.

### 2.4. Volatile Organic Compounds Analysis

#### 2.4.1. The Electronic Nose (E-Nose) Analysis

A total of 2 g of the sample was transferred into a headspace vial (20 mL) and then equilibrated at 25 °C for 30 min [20]. Subsequently, the sample was analyzed using an E-nose system (Shengbao CNose-18, Shanghai, China), which comprises 18 sensors. Detailed descriptions of the sensors can be found in Table S1. The analysis conditions were as follows: sampling duration, 120 s; airflow rate, 1 L/min; purging time, 120 s. Each sample group was tested six times.

#### 2.4.2. GC-IMS Analysis

The volatile flavor compounds of CPs and COs were analyzed though the GC-IMS method [21]. Initially, 2 g of the sample was incubated at 80 °C for 20 min in a 20 mL headspace. The FlavourSpec^®^ instrument (G.A.S., Dortmund, Germany) was employed for VOC detection, with data analysis and visualization conducted using the functional software developed by G.A.S., while compound identification was achieved by the RI and IMS databases. The injection conditions were as follows: injection method, headspace injection, splitless mode; injection volume, 500 µL; injector temperature, 85 °C; heating method, oscillation heating; cleaning time, 5 min. The GC parameters were as follows: column, MXT-5 (RESTEK Corporation, Bellefonte, PA, USA); column temperature, 60 °C; carrier gas, high-purity N_2_ (purity ≥ 99.999%); run time, 30 min. The temperature program was configured with the following gradient: the initial carrier gas flow at 2 mL/min, followed by sequential linear increased to 10 mL/min (held for 5 min), followed by linear increased to 15 mL/min (held for 10 min), 50 mL/min (held for 5 min), and 100 mL/min (held for 10 min). For IMS analysis: operational conditions included a tritium-based ionization source and a 9.80 cm drift tube. Ultrapurity nitrogen (≥99.999%) served dual roles as carrier and drift gas, with the drift chamber thermostated at 45 °C with a gas flow of 150 mL min^−1^.

#### 2.4.3. HS-SPME-GC-MS Analysis

HS-SPME-GC-MS was employed for VOCs determination [22]. For compound extraction, 200 mg of each sample was weighed into a 20 mL headspace vial, after which 10 μL of 2-octanol (10 mg/L stock in dH_2_O) was spiked in as an internal standard.

GC-MS Analysis: The HS-SPME procedure was conducted by a PAL rail system with the following parameters: incubation temperature, 60 °C; preheat time, 15 min; incubation time, 30 min; desorption time, 4 min. Volatile separation and identification were conducted with an Agilent 7890B/5977B (Agilent, Palo Alto, CA, USA) GC-MS system equipped with a polar DB-Wax capillary column (30 m × 250 μm × 0.25 μm, Agilent, Palo Alto, CA, USA). The system was configured in splitless injection mode with helium carrier gas, maintaining a front inlet purge flow of 3 mL/min and a column flow rate of 1 mL/min. The temperature gradient was programmed as follows: initial isothermal hold at 40 °C for 4 min, followed by a ramp to 245 °C at 5 °C/min with a final 5 min hold. Temperatures were configured as follows: injector 250 °C, transfer line 250 °C, ion source 230 °C, and quadrupole 150 °C. MS detection employed electron impact ionization (−70 eV) in scan mode (*m*/*z* 20–400) with 2.37 min solvent delay. Chroma TOF 4.3X software (LECO) enabled comprehensive data processing, including peak extraction, baseline adjustment, alignment, deconvolution, and compound identification based on retention indices (RI) and the NIST database. Compound identification required a retention index (RI) deviation within ±10 units, compared to authentic standards or published literature values. The relative content of each compound was calculated using the internal standard normalization method based on peak area.

#### 2.4.4. Identification of Relative Odor Activity Values (ROAVs)

*ROAVs* were used to assess the key aroma components in CPs and COs. The component with highest contribution to the flavor of sample were defined as 100, the ROAV_A_ values of other components were obtained by the following Equation (2) [5,23].

(2) $$ROVAs\;=\;100\;\times \;\frac{{C\%}_{A}\;\times \;T_{stan}}{C_{\%stan}\;\times \;T_{A}}$$

Note: *C*%*_A_* and *T_A_* were the relative content (%) and threshold (μg/kg) of each aroma component; *C*%*_stan_* and *T_stan_* were the relative content (%) and threshold (μg/kg) of each aroma component that contributed the most to the aroma of the sample.

### 2.5. LC-MS/MS for Non-Volatile Flavor Compounds Analysis

Sample preparation followed a modified version of the method described [21]. Briefly, samples were mixed with extraction solution (MeOH: ACN: H_2_O, 2:2:1 (*v*/*v*/*v*)) supplemented with deuterated internal standards and beads. The extraction protocol included mechanical homogenization (35 Hz, 4 min), triple sonication (5 min per cycle, 4 °C water bath), protein precipitation (−40 °C, 1 h), and centrifugation (12,000 rpm, 15 min, 4 °C). The resulting supernatant was collected for subsequent LC-MS/MS analysis.

LC-MS/MS was performed on a Vanquish UHPLC-Orbitrap Exploris 120 platform (Thermo Fisher Scientific, Waltham, MA, USA). Separation was carried out with a binary eluent system: aqueous 25 mM ammonium acetate–ammonium hydroxide adjusted to pH 9.75 (solvent A) versus acetonitrile (solvent B). The auto-sampler temperature was 4 °C, and the injection volume was 2 uL. MS/MS detection utilized information-dependent acquisition (IDA) mode with electrospray ionization (ESI) under optimized parameters: sheath gas, 50 Arb; auxiliary gas, 15 Arb; capillary temperature, 320 °C; full MS resolution, 60,000; MS/MS resolution, 15,000. Data processing involved mzXML conversion, feature detection, extraction, alignment, and integration using ProteoWizard, R-based in-house program, and BiotreeDB (V3.0) for metabolite identification.

### 2.6. Sensory Evaluation

The sensory characteristics of chili powders and chili oils were evaluated following the established method with slight modifications [24]. A trained sensory panel (n = 12, 6 males and 6 females, aged 20–28 years) was established, comprising members who had no affinity for spicy foods, no adverse reactions to pungency, no allergies to spicy substances, and prior sensory evaluation experience. They were fully informed of the purpose of the study and provided written consent to participate in the experiment. The study protocol was approved by the Ethics Committee of Sichuan Agricultural University to ensure adherence to ethical standards throughout the research. Participants underwent two preliminary training sessions to familiarize themselves with the evaluation criteria (color, aroma, and taste) and scoring system. Reference samples (e.g., unprocessed dried chili powder and commercial chili oil) were used to calibrate perception consistency across panelists. Place the chili powders (1 g) and chili oils (5 g) from different treatment groups in food-grade plastic cups for smelling and tasting. Evaluations were conducted in individual sensory booths at room temperature. Panelists assessed chili powder and chili oil separately in randomized order, with a 5 min interval between samples to avoid fatigue. The specific scoring details were provided in Supplementary Tables S2 and S3. A 10-point scale was used to evaluate five sensory attributes: color, rapeseed chili flavor, bitterness/off-flavor, spiciness, and overall aroma for chili powder; color, rapeseed oil flavor, bitterness/off-flavor, spiciness, and overall aroma for chili oil. Scores for each attribute were categorized into three tiers: low (1–4), medium (5–7), and high (8–10). The overall acceptability was defined as the sum of the scores for all five evaluation criteria. Purified water was provided for palate cleaning. Each sample was tested in three independent evaluations, and the final score was expressed as the arithmetic mean of these replicates.

### 2.7. Statistical Analysis

A factorial design was employed to investigate the effects of thermal treatment (stir-frying, microwaving, and oven-roasting) on chili powder and oil flavor profiles. Unless specified, experiments were carried out in triplicate, with results reported as mean ± standard deviation. Statistical analysis was conducted using SPSS software (version 16.0 for Windows), employing one-way analysis of variance (ANOVA) followed by Tukey’s test, with *p*-values < 0.05 considered statistically significant. Orthogonal partial least squares discriminant analysis (OPLS-DA) was performed using SIMCA 14.1 (Umetrics, Umeå, Sweden) to assess sample groupings. The reliability of the model was assessed through a 200-cycle permutation test. Due to the ordinal nature of the sensory scoring data, non-parametric statistical methods were employed. GraphPad Prism (version 7) and origin 2018 were used to data visualization.

## 3. Result and Discussion

### 3.1. Analysis of Color

Color changes could reflect the extent of Maillard reactions and browning in chili product and the quality of chili oil [10]. L* represents brightness, while a* and b* indicate red–green and yellow–blue balance, respectively [19]. Table 1 illustrated the color changes in chili powders and oils. Thermal treatments significantly increased the a* and b* values of SC, OC, and MC (*p* < 0.05). The color of chili peppers primarily derived from carotenoids such as capsanthin, capsorubin, and zeaxanthin, whose degradation influenced a* and b* values [25]. A brief exposure to high temperature might have disrupted the cellular structure of capsorubin, capsanthin, and zeaxanthin, thereby enhancing pigment release from chili peppers and consequently increasing a* and b* values [25,26]. Moreover, thermal processing induced Maillard reactions between reducing sugars and amino acids in chili peppers, generating brown compounds that decreased L* values and altered a* and b* values [1].

Furthermore, color changes in CPs affected COs, as evidenced by increased a* and b* values, and decreased L* values in SCO, OCO, and MCO (*p* < 0.05). Further heating of pre-processed CPs at 180 °C in rapeseed oil intensified the Maillard reaction, producing additional brown compounds (melanoid) that reduced L* values and increased b* values in SCO, OCO, and MCO (*p* < 0.05).

### 3.2. Analysis of Volatile Organic Compounds

#### 3.2.1. Analysis of E-Nose

**Figure 1:**
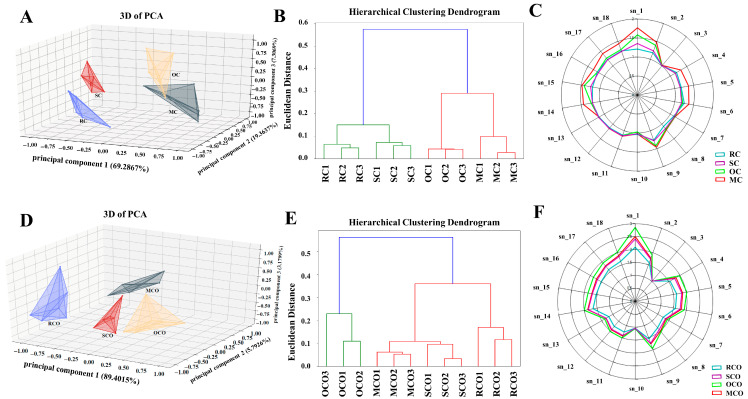
E-nose analysis of chili powders and chili oils. Three-dimensional PCA diagram (n = 6) (**A**), hierarchical clustering dendrogram (**B**), and the radar map (**C**) of chili powders; 3D PCA diagram (n = 6) (**D**), hierarchical clustering dendrogram (**E**), and the radar map (**F**) of chili oils.

Minor differences in VOCs elicited differential responses in E-nose sensors sensitive to aroma profiles. Principal component analysis (PCA) in three-dimensional space revealed cumulative variance contributions of 95.96% (CPs) and 98.37% (COs) for the first three principal components (Figure 1A,D). Distinct separation among RC, SC, OC, MC, and their corresponding chili oils (RCO, SCO, OCO, MCO) indicated significant volatile composition differences. Hierarchical clustering analysis further validated that SC, OC, and MC showed distinct VOCs profiles from RC (Figure 1B). After secondary activation to produce chili oil, OCO exhibited the most significant differences from other groups, and MCO, SCO, and RCO also showed certain differences (Figure 1E). Radar plots of 18 metal oxide sensor responses (Figure 1C,F; Table S1) showed comparable VOC categories across groups but significant intensity variations in specific sensors (Sn_1, Sn_2, Sn_4, Sn_5, Sn_6, Sn_9, Sn_14, Sn_15). Sensor Sn_6 exhibited high sensitivity to aldehydes and ketones, while Sn_9 was particularly responsive to alkanes, alcohols, and ketones, and Sn_15 showed broad sensitivity to a range of compounds, including carbonaceous substances, alcohols, and aldehydes. Specifically, the sensor signals followed MC > OC > SC > RC (Figure 1C) and OCO > MCO > SCO > RCO (Figure 1F), implying that thermal processing altered key flavor components (propane, carbonaceous compounds, aldehydes/ketones, alkanes, alcohols, and toluene/acetone/ethanol). Li et al. [5] reported that aldehydes, alcohols, and ketones were primary chili VOCs. However, our results suggested that thermal treatments modify concentrations rather than profile categories.

Although E-nose effectively differentiated overall flavor patterns, its limitations in specific VOCs’ identification necessitated complementary analyses [27]. Consequently, comprehensive characterization was subsequently performed using GC-IMS and GC-MS techniques.

#### 3.2.2. Analysis of GC-IMS

GC-IMS integrates the superior separation efficiency of GC with the enhanced sensitivity of IMS, enabling rapid and accurate detection of volatile and semi-volatile compounds [28]. A total of 72 VOCs were determined by the retention indices (RI), retention times (Rt), and drift times (Dt) of the compounds, in accordance with the NIST and IMS databases integrated within the GC×IMS system. These VOCs included 23 aldehydes, 15 alcohols, 11 esters, 8 ketones, 4 hydrocarbons, 8 heterocycles, and 3 other compounds (Tables S4 and S5).

#### Topographic Map Analysis

**Figure 2:**
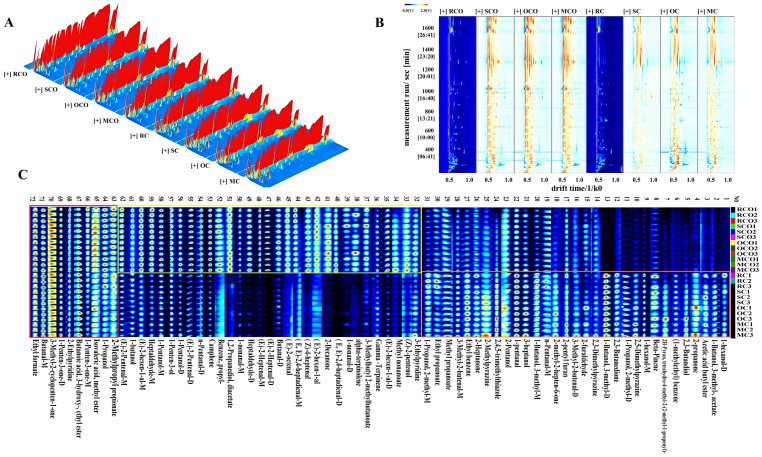
GC-IMS analysis of flavor profile changes in chili powders and chili oils with different thermal treatments. Three-dimensional-topographic (**A**); 2D topographic plots (**B**); fingerprint of volatile compounds (**C**). Orange-boxed region: No. 1–31; Yellow-boxed regions: No. 32–62; Red-boxed regions: No. 63–72.

Spectral differences among samples were directly compared in Figure 2. Three-dimensional topographic plots revealed stronger signal intensities in heat-processed chili powders (SC, OC, MC) and their corresponding oils (SCO, OCO, MCO) compared to raw controls (RC and RCO) (Figure 2A). To enhance data comparability, reactant ion peak (RIP) and ion migration time were normalized, with differential visualization presented in 2D topographic plots (Figure 2B). The vertical axis represents GC retention time (Rt), and the horizontal axis indicates ion mobility drift times (Dt), with the red vertical line at Dt 0.5 denoting the normalized reactant ion peak (RIP). Each dot to the right of RIP corresponds to a VOC, where color saturation reflects compound concentration level (white: low; red: high) [29]. The spectra of the control groups (RC and RCO) were used as references, and differential spectra were generated by subtraction. Uniform signal intensities appear white, while red and blue regions, respectively, indicate higher and lower concentrations relative to controls, with color depth reflecting magnitude of difference [30,31]. Compared to RC, SC, OC, and MC (particularly OC and MC) displayed extensive red regions, indicating a significantly increased compound concentration, which is consistent with the results from the E-nose analysis. The signal intensities of several aldehydes (e.g., (E,E)-2,4-heptadienal-M, (E)-2-hexenal-M, (E)-2-pentenal-D, and (Z)-4-heptenal), esters (e.g., methyl nonanoate), hydrocarbons (e.g., α-terpinolene, camphene), and heterocyclic compounds (e.g., 2-methylpyrazine, 2,5-dimethylpyrazine, 2-ethylpyridine) were significantly enhanced (*p* < 0.05, Table S4). Similarly, SCO, OCO, and MCO showed enhanced VOCs diversity compared to RCO. The observed enhancement in VOCs signals was primarily attributable to increased concentrations of several aldehydes (e.g., (E,E)-2,4-heptadienal-D, (E)-2-hexenal-D, butanal-D, 2-furaldehyde, 3-methyl-2-butenal-M, n-pentanal-M), ketones (e.g., 2-decanone), and heterocyclic compounds (e.g., 2-methylpyrazine, 3-ethylpyridine, 2,4,5-trimethylthiazole) (*p* < 0.05, Table S5). These changes likely originated from thermally induced structural modifications (e.g., cellular matrix disruption facilitating compound release) and chemical transformations including oxidation, pyrolysis, and Maillard reactions. For instance, unsaturated fatty acids form volatile aldehydes (e.g., nonanal, hexanal) via β-scission or alcohols (e.g., 1-octen-3-ol, 1-nonanol) via reduction during high-temperature, radical-mediated hydroperoxide decomposition [32]. Moreover, thermal processing further decomposed non-volatile precursors (e.g., amino acids and reducing sugars) into volatile aroma compounds (e.g., furans) through Maillard-derived heterocyclic compound formation [33].

#### Fingerprint Analysis of Differences in VOCs in CPs and COs

To further elucidate the differential effects of thermal processing on VOCs in CPs and COs, characteristic fingerprint spectra were established (Figure 2C). These fingerprints enabled comparative evaluation of VOC profiles through signal intensity variations (increase, decrease, or disappearance) [34].

The orange-boxed region (No. 1–31) was mainly present in CPs and was scarcely detected in COs, indicating transformation or loss during secondary heating. Compared to RC, higher levels of acetic acid butyl ester, 2-propanone, 2,3-butanediol, 2,5-dimethylpyrazine, and 2-methylpyrazine were found in SC, OC, and MC, which contribute fruity, spicy, nutty, and roasted aromas [35]. 2-Propanone was a common product of lipid oxidation and thermal degradation of sugars or organic acids, while 2,5-dimethylpyrazine and 2-methylpyrazine were direct products of the Maillard reaction. Higher concentrations of 2-furaldehyde, 2,5-dimethylpyrazine, 2-pentyl furan, 2-methylpyrazine, and 2,3-dimethylpyrazine were detected in OC and MC. These compounds, typical products of the Maillard reaction, imparted rich caramel, roasted, almond, and nutty aromas [36]. RC showed higher concentrations of 1-hexanol-M, n-pentanal-M, and 2-methyl-1-propanol-M. Research demonstrated that most alcohols, especially low-molecular-weight alcohols, consistently decreased during drying due to their high volatility and evaporation [37].

The yellow-boxed regions (No. 32–62) predominantly contained VOCs that were mainly found in COs. Alcohols and aldehydes (e.g., 1-butanol, 1-nonanal-D, (E)-2-heptenal, butanal-D, and (E)-2-hexen-1-al-M) and hydrocarbons (e.g., gamma-terpinene, alpha-terpinolene, and camphene) were the main characteristic flavor compounds, which are consistent with the reported research results [5]. Higher concentrations of (E)-2-hexen-1-al-D, (Z)-2-pentenol, 2-decanone, (Z)-4-heptenal, and heptanaldehyde-D were detected in SCO, OCO, and MCO, providing more pleasant grassy, fruity, nutty, and fatty aromas.

The red-boxed regions (No. 63–72) represented shared compounds with comparable intensities in both CPs and COs, including ethyl formate, butanal-M, 1-penten-3-one-D, 1-propanol, 2-ethylpyridine, and 3-hydroxybutanoic acid ethyl ester. These compounds were relatively stable, and their concentrations did not change significantly, despite different thermal treatments. These results indicated that during the process of converting chili powder into chili oil, the flavor underwent significant changes. Appropriate heat treatment and secondary activation had a positive impact on flavor retention, thereby influencing the final flavor characteristics of CPs and COs.

#### 3.2.3. Analysis of HS-SPME-GC-MS

A total of 220 and 207 volatile compounds were identified in CPs and COs, respectively. These VOCs included alcohols (38 vs. 33), aldehydes (26 vs. 25), acids (23 vs. 25), hydrocarbons (42 vs. 32), ketones (23 vs. 28), heterocycles (32 vs. 40), esters (29 vs. 13), and others (7 vs. 11) (Tables S6 and S7). Quantitative analysis of the total relative contents revealed that the levels of aldehydes, acids, hydrocarbons, ketones, heterocycles, and esters increased in SC, OC, and MC. In chili oil, higher contents of aldehydes, ketones, and heterocycles were observed in SCO, OCO, and MCO. These results were in agreement with the trend observed in the E-nose radar plot, where Sn_6, Sn_9, and Sn_15 showed enhanced signals (Figure 1C,F), demonstrating that heat treatment significantly promotes the release of flavor compounds.

Alcohols are derived from the cleavage of aliphatic carbon chains, oxidation of olefins, and microbial metabolism [38]. Although alcohols are generally considered to have limited contribution to the overall flavor profile due to their high odor thresholds, their potential synergistic effects within complex food matrices are often overlooked. Wang et al. found that glycerol, an abundant polyol in Baijiu, significantly enhanced the release of key aroma compounds such as dimethyl trisulfide and 3-methyl-1-butanol [39]. Furthermore, glycerol formed hydrogen-bonded complexes with other flavor constituents, thereby modulating their perceived aroma intensity either through enhancement or suppression [39]. The major alcohols identified in CPs include ethanol, 1-hexanol, phenylethyl alcohol, 1-pentanol, 4-methyl-1-pentanol, and 3-methyl-1-butanol, which were consistent with those reported in linear, millet, and bullet peppers [1]. We observed that certain alcohols decreased in content after thermal treatment, such as 1-hexanol, 1-pentanol, 4-methyl-2-propanol, 1-methoxy-2-propanol, and 2-heptanol. This reduction may attributed to their volatility and esterification under high-temperature conditions [40]. On the other hand, higher concentrations of 2-furanmethanol, 5-methyl-2-furanmethanol, and furaneol were detected in OC and MC. The formation of these compounds was associated with the Maillard reaction, 5-hydroxymethylfurfural, and lipid co-oxidation. For example, pentoses (such as ribose and xylose) react with amino acids via the Maillard reaction to produce furfural (2-furaldehyde), which is subsequently reduced to 2-furanmethanol through thermal or enzymatic reduction processes [41]. In COs, the top five alcohols in terms of content were ethanol, 2-furanmethanol, furaneol, 5-methyl-2-furanmethanol, and 1-penten-3-ol. Except for ethanol, the contents of these major alcohols significantly increased in SCO, OCO, and MCO, especially in OCO. The Maillard reaction serves as a critical pathway for the formation of 2-furanmethanol and its methylated derivatives (e.g., 5-methyl-2-furanmethanol) [42].

Aldehydes are formed through lipid oxidation, protein degradation, and via decarboxylation or deamidation of amino acids, critically influencing chili oil flavor profiles by providing the fat and beef flavor [21,43]. Branched-chain amino acids (e.g., leucine and isoleucine) undergo non-enzymatic oxidative decarboxylation to form characteristic aldehydes such as 3-methylbutanal and 2-methylbutanal [44]. Additionally, under thermal conditions, reactive carbonyls from lipid oxidation participate in Strecker degradation with α-amino acids, leading to aldehyde formation. Both CPs and COs exhibited marked increases in aldehyde content following thermal processing, with hierarchical trends of RC < SC < MC < OC and RCO < MCO < SCO < OCO. The major aldehydes identified in CPs and COs include furfural, benzeneacetaldehyde, benzaldehyde, 3-methyl-butanal, and (E,E)-2,4-heptadienal. Benzeneacetaldehyde and benzaldehyde can be obtained by Streker degradation [13]. Notably, (E,E)-2,4-heptadienal was identified as the most abundant aldehyde in chili oils, which contributed grassy and fruity nuances, corroborating its role as a key aroma-active compound [5].

Heterocycles are pivotal in the flavor development of chili products [32,45]. The Maillard reaction serves as a principal mechanism for the generation of heterocyclic compounds, initially involving the condensation between reducing sugars and amino acids and subsequent Strecker degradation, where α-dicarbonyl compounds react with amino acids to produce aldehydes and aminoketones [33]. These intermediates subsequently condense to form furans, pyrazines, pyridine, and other nitrogen-containing heterocycles. Additionally, pyrans and furans can be generated through sugar dehydration and thermal degradation (particularly under high temperatures) as well as through secondary pathways of lipid oxidation [46]. Characteristic Maillard-derived pyrazines and furans, such as tetramethylpyrazine, 2,6-dimethylpyrazine, and 2-pentylfuran, contribute significantly to roasted and nutty aroma profiles in thermally processed foods. OC and OCO exhibited the highest levels of these compounds, indicating that oven treatment had the most significant impact on chili flavor. Similarly, furan derivatives, such as 2-pentylfuran, which ranks second in content among COs, also reflect the intensity of the reaction. In low-moisture substrates, the production of roasted malt-related heterocyclic aromatic compounds sharply increases with rising process temperatures [47].

Certain hydrocarbons (such as p-cymene and styrene), ketones (such as acetoin and 1-hydroxy-2-propanone), and esters (such as ethyl acetate and methyl acetate) imparted fruity, floral, and lipid-like aromas, as well as bitter and pleasant odors to chili products. As the highest proportion among hydrocarbons, alkanes exhibit elevated flavor thresholds with minimal aromatic distinctiveness. Consequently, they have a limited impact on the overall aroma profile of chili peppers [48].

#### Analysis of Key Aroma-Active Volatiles

The relative odor activity value (ROAV) was employed to evaluate the contribution of volatile compounds to the overall flavor profile, thereby identifying key aroma-active compounds. Compounds with ROAV ≥ 1 were defined as key aroma contributors. A total of 29 key odorants (ROAV ≥ 1) were identified in this study (Table S8). In CPs, ethyl 2-methylbutanoate was identified as the most potent odorant in the RC, with its ROAV set to 100. 2-methylbutanal, which contributed more to the overall flavor, was set to 100 in SC, OC, and MC. In COs, benzeneacetaldehyde was the dominant aroma contributor in RCO and MCO (ROAV = 100), while ethyl 2-methylbutanoate showed the highest ROAV in SCO and OCO (ROAV = 100). There were 21, 19, 15, and 19 key flavor compounds in RC, SC, OC, and MC, respectively; and 17, 14, 13, and 14 key flavor compounds in RCO, SCO, OCO, and MCO, respectively.

Among these substances, aldehyde was the most abundant, such as 2-methyl-butanal (ROAVs: 47.83–100), benzeneacetaldehyde (ROAVs: 27.56–100), benzaldehyde (ROAVs: 4.51–33.91), nonanal (ROAVs: 8.81–45.58), and octanal (ROAVs: 1.96–5.08), indicating their crucial role in shaping the aroma profile of chili products. Notably, benzeneacetaldehyde and 2-methylbutanal were the most important shared aroma compounds in both CPs and COs. 2-methylbutanal, derived from the Strecker degradation of isoleucine, contributed nutty, cocoa-like, and fruity notes to the overall aroma. Additionally, nonanal (ROAVs: 26.40–31.46), heptanal (ROAVs: 4.32–6.60), (E)-2-pentenal (ROAVs: 4.57–6.04), and hexanal (ROAVs: 3.52–5.00) were identified as key aroma compounds in COs, imparting distinctive fatty, floral, and fruity notes. 1-Octen-3-ol (ROAVs: 1.57–14.53), a product of linoleic acid oxidation, contributed mushroom, rose, and hay-like aromas and played a non-negligible role in the chili flavor profile [5]. Other contributors, including 2-methylbutanoic acid, 2,3-butanedione, 2-pentylfuran, and 2-methoxy-4-vinylphenol, also played significant roles. In addition, some key aroma compounds in CPs, such as 1-hexanol, octanal, furfural, and 3-methylbutanoic acid, exhibited ROAV < 1 in OC, suggesting a potential shift in the aroma profile of OC compared to other samples.

To elucidate the impact of different thermal treatments on chili peppers, subsequent analyses will focus separately on the results of CPs and COs.

#### Influence of Different Thermal Treatments on the Flavor of CPs

**Figure 3:**
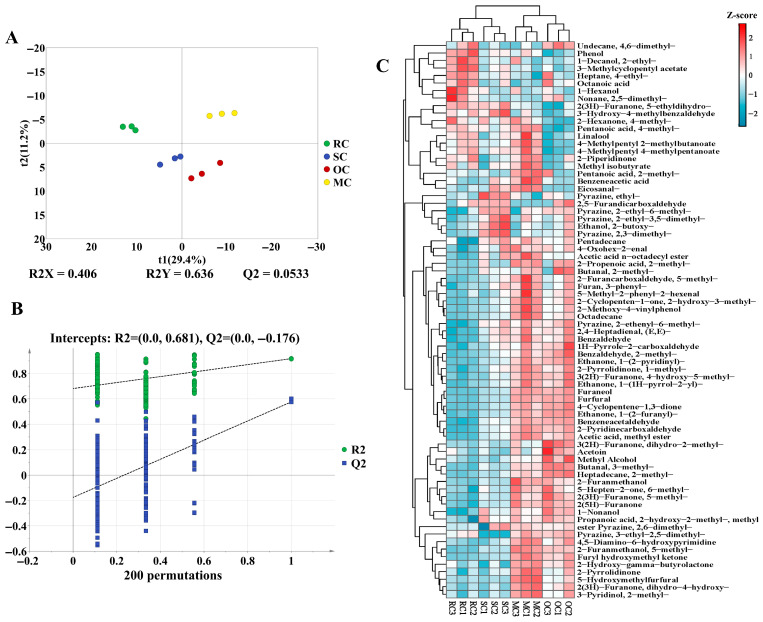
GC-MS flavor compounds analyses of chili powders. OPLS-DA score map (**A**); OPLS-DA permutation test (**B**); hierarchical clustering heatmap of differential flavor compounds (**C**). R^2^ (goodness-of-fit) indicates the proportion of variance explained by the model, with its line showing how fit changes with complexity. Q^2^ (predictive ability) measures cross-validated predictive variance, and its line helps evaluate generalization.

The orthogonal partial least squares-discriminant analysis (OPLS-DA) models were constructed to discriminate between different sample groups (Figure 3A). Principal components PC1 and PC2 explained 29.4% and 11.2% of total variance, respectively. Clear separation of thermally processed groups (RC, SC, OC, MC) across quadrants confirmed significant divergence in volatile profiles. Furthermore, the OPLS-DA model was validated using a permutation test with 200 permutations (Figure 3B). The intercepts of R^2^ and Q^2^ were 0.681 and −0.176, respectively, indicating that the models were robust and not overfitted.

In this study, one-way ANOVA was employed to identify differential flavor compounds, with *p* < 0.05 considered statistically significant. A total of 74 differential flavor compounds were identified through comprehensive analysis and visualized using a clustering heatmap (Figure 3C). Overall, the heatmap revealed distinct clustering patterns among the samples, indicating that different processing methods significantly influenced the volatile compound profiles. 2-Ethyl-1-decanol, 3-dethylcyclopentyl acetate, 4-ethyl-heptane, octanoic acid, and 1-hexanol were identified as differential flavor compounds in RC. Octanoic acid typically exhibited a sweat-like, waxy, rancid, and unpleasant irritating odor, while 1-hexanol was characterized by resinous, fusel, and fatty notes [1]. The decrease in the content of these compounds in SC, OC, and MC might be attributed to volatility or chemical reactions with other components during thermal processing, indicating that appropriate heat treatment could reduce these unpleasant odors. Maillard reaction products, such as ethyl pyrazine, 2-ethyl-6-methylpyrazine, and 2,3-dimethylpyrazine were highly expressed in SC, which contributed to butter, peanut, cocoa, and nutty flavors. Notably, compared to RC, multiple compounds were upregulated in OC and MC, including 1-nonanol, 2-furanmethanol, (E,E)-2,4-heptadienal, 3-methylbutanal, benzaldehyde, benzeneacetaldehyde, furfural, octadecane, and acetic acid methyl ester. 2-Furanmethanol, and acetic acid methyl ester collectively impart complex aromatic profiles, which formed via slower esterification reactions and aldehyde oxidations, characterized by faint alcoholic, sweet, and pleasant fruity odors [49]. These compounds contribute to a richer flavor profile in chili products. Specifically, thermal treatment can intensify the Maillard reaction, promote lipid oxidation and hydrolysis, and thereby facilitate the release and transformation of flavor compounds in chili.

#### Influence of Secondary Heat Activation on the Flavor of COs

**Figure 4:**
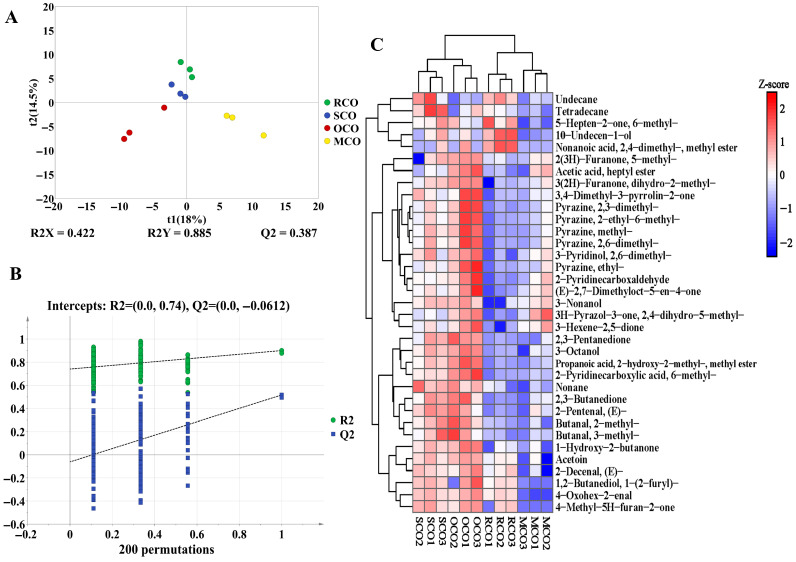
GC-MS flavor compounds analyses of chili oils. OPLS-DA score map (**A**); OPLS-DA permutation test (**B**); hierarchical clustering heatmap of differential flavor compounds (**C**). R^2^ (goodness-of-fit) indicates the proportion of variance explained by the model, with its line showing how fit changes with complexity. Q^2^ (predictive ability) measures cross-validated predictive variance, and its line helps evaluate generalization.

OPLS-DA effectively separated RCO, SCO, OCO, and MCO into four distinct groups (Figure 4A), with the principal components PC1 and PC2 accounting for 18% and 14.5% of the total variance, respectively. Permutation test showed that the intercepts of R^2^ and Q^2^ were 0.74 and −0.0612, respectively, indicating that the models were robust and not overfitted (Figure 4B).

Hierarchical clustering of 35 key differential flavor compounds further visualized compositional disparities among COs (Figure 4C). The results showed that most flavor compounds in SCO and OCO were significantly upregulated, with higher level of certain aldehydes (e.g., 2-methylbutanal, 3-methylbutanal, 3-octanol, and 3-nonanol), ketones (e.g., 2,3-pentanedione, (E)-2,7-dimethyloct-5-en-4-one, and acetoin), and heterocyclic compounds (e.g., 2,3-dimethylpyrazine, dimethylpyrazine, ethylpyrazine, and 2,6-dimethylpyrazine), indicating richer flavor compounds and a more intense aroma. 2-Methylbutanal and 3-methylbutanal were primarily derived from the Strecker degradation, enzymatic reactions, and oxidation of leucine and isoleucine, and played a significant role in promoting fruity and nutty aromas [50]. Furthermore, consistent with the findings in OC, the differential aroma compounds in OCO were predominantly associated with thermal degradation and Maillard reaction products, such as furans and pyrazines, indicating that prolonged roasting effectively promoted these reactions.

### 3.3. Analysis of LC-MS/MS

The non-volatile components in CPs and COs were profiled using untargeted LC-MS/MS. Data acquisition was performed in both positive (POS) and negative (NEG) ESI modes. A total of 2251 and 1978 non-volatile compounds were identified in CPs and COs, respectively, under these two modes (Tables S9 and S10).

#### 3.3.1. Influence of Different Thermal Treatments on the Capsaicinoids

Capsaicinoids, a group of bioactive vanilloids, are pivotal in imparting the unique pungent flavor of chili peppers, primarily composed of capsaicin and dihydrocapsaicin (comprising 80–90% of the total capsaicinoid content), with nordihydrocapsaicin and homodihydrocapsaicin I also contributing to spiciness [51,52]. As shown in Figure S1A,B, the content of capsaicinoids in CPs and COs had no obvious change (*p* > 0.05). Previous studies demonstrated that capsaicinoids showed no significant change when heated continuously at 150 °C [53,54]. In this study, the CPs underwent relatively mild treatments of either brief heating (SC and OC: 3 min) or low-temperature processing (100 °C for OC). Although these conditions disrupted cellular structures and facilitated the release of volatile compounds, they remained well below the threshold required for capsaicinoid degradation. Furthermore, appropriate heating temperatures enhanced capsaicinoid stability through peroxidase inactivation and the suppression of non-enzymatic degradation pathways [13,55]. Likewise, RCO, SCO, OCO, and MCO were all subjected to a second thermal activation at the same oil temperature (180 °C) and consequently exhibited consistent variation trends.

#### 3.3.2. Influence of Different Thermal Treatments on the Non-Volatile Compound Profiles

**Figure 5:**
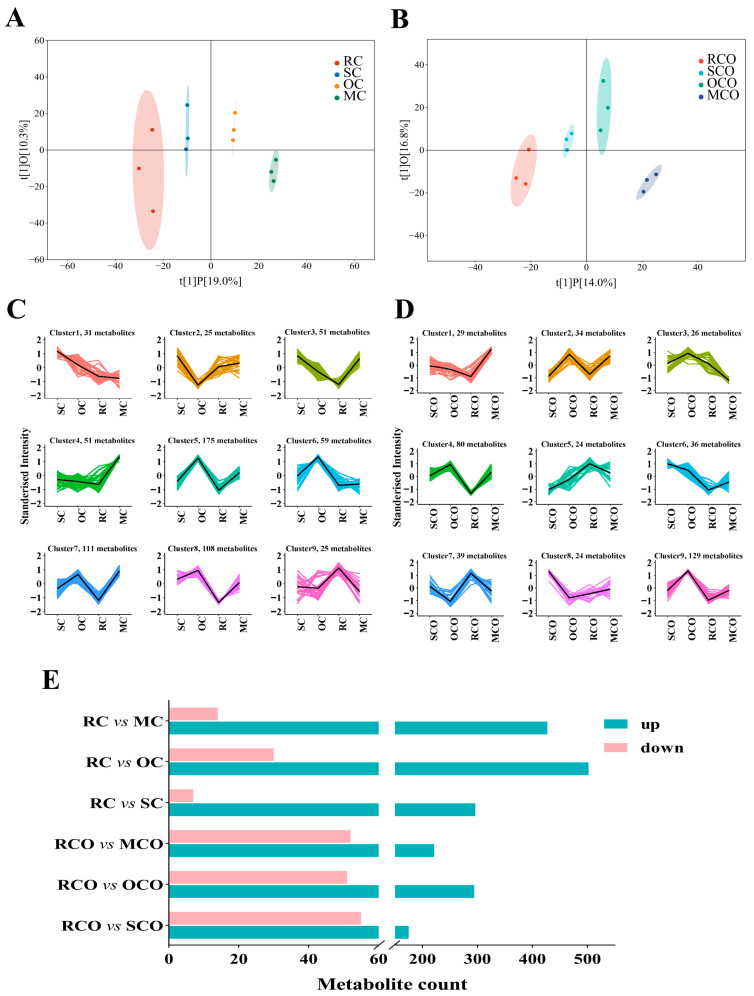
LC-MS/MS analyses of chili powders and chili oils. OPLS-DA score map of chili powders (**A**), and chili oils (**B**); K-mean diagram of chili powders (**C**), and chili oils (**D**); the statistical histogram of differential compounds in each group (**E**).

OPLS-DA was subsequently applied to these untargeted compounds (Figure 5A,B). Both CPs and COs were classified into four distinct clusters and revealed cumulative variance contributions of 29.3% and 30.8%, respectively. The results showed that different thermal treatments significantly altered the non-volatile compound profiles of chili products. Furthermore, these heat-induced compositional changes persisted and influenced the final composition of processed chili oils. Differential compounds were identified in CPs using a significance threshold of *p* < 0.05, yielding 631 significantly altered compounds (Figure 5C). These compounds were, respectively, classified into nine distinct clusters through hierarchical clustering analysis, revealing substantial impacts of thermal processing methods on samples. Compared to RC, nearly all compounds across clusters demonstrated varying degrees of upregulation after thermal treatments, with the exception of cluster 9 in CPs (Figure 5C). As shown in Figure 5D, 421 differential compounds were identified in COs (*p* < 0.05). SCO, OCO, and MCO groups exhibited higher metabolite abundance compared to RCO, except for cluster 5 and 7 compounds, which showed downregulation.

To further elucidate thermal processing effects, differential feature regulation patterns were systematically analyzed between experimental groups and controls (Figure 5E). Thermal treatment coupled with secondary thermal excitation significantly increased the number of upregulated compounds, leading to a drastic change in compound composition and enriching the flavor of chili products. Notably, LC-MS/MS findings aligned with GC-MS results, demonstrating that oven-baked and microwaved treatments enhanced the release of flavor and taste compounds. This synergistic effect between thermal modalities resulted in more sophisticated flavor profiles in both chili powders and their corresponding oils.

### 3.4. Analysis of Sensory Evaluation

**Figure 6:**
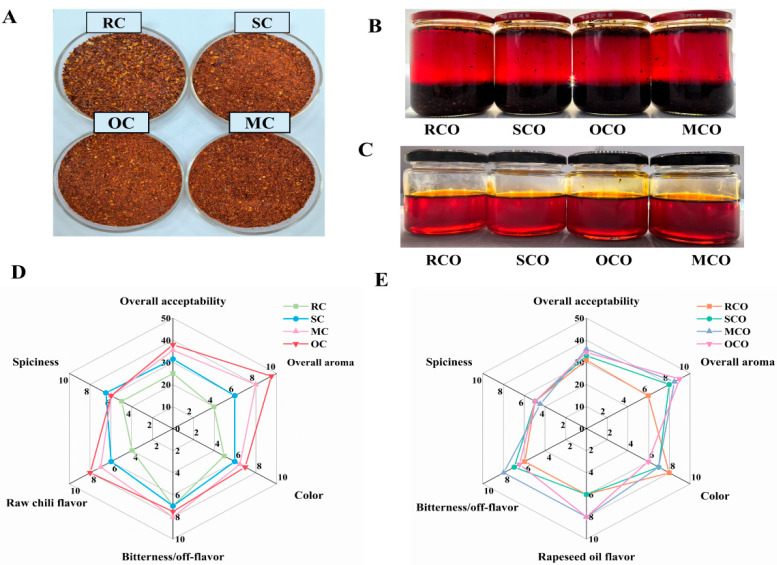
Visual and sensory evaluation comparison of chili powders and chili oils. Chili powders after different thermal treatments (**A**); chili oils prepared from chili powders (**B**); the filtered chili oils (**C**); sensory evaluation radar map of chili powders (**D**), and chili oils (**E**).

The sensory quality and flavor of products play pivotal roles in consumer acceptance. Figure 6A illustrated the color and texture of CPs produced after various thermal treatments. The visual color intensity ranking (OC > MC > SC > RC) indicated that thermal processing enhanced the release of red pigments. Previous studies have demonstrated that the a* value of dried red chili peppers significantly increased due to the change in chlorophyll and carotenoids as the blanching time extended [3]. Comparative color analyses of COs containing residual chili particles (Figure 6B) and filtered oils (Figure 6C) revealed distinct chromatic profiles: RCO displayed higher clarity, while OCO and MCO exhibited darker hues, consistent with color analysis (Table 1). SCO, OCO, and MCO underwent two thermal reactions, triggering the Maillard reaction and producing more browning compounds.

The sensory evaluation depicted in Figure 6D,E revealed that RC and RCO had the lowest overall acceptability scores, whereas OC and its derivative oil OCO achieved the highest sensory scores and overall aroma. This may be attributed to the oven-baked treatment significantly enhancing the release of pleasant volatile compounds, such as (E,E)-2,4-heptadienal, benzaldehyde, 2-methylbutanal, 3-methylbutanal, furfural, 2,3-butanedione, acetic acid, and methyl ester, which contribute floral, fruity, nutty, and fatty aromas. OC, SC, and MC enhanced the overall aroma and color of chili peppers, diminished the raw chili flavor, and this impact persisted in enhancing the sensory scores of chili oil. The observed decrease in hexanal, characterized by its leafy, fresh, and green-grass aroma following thermal treatment, was likely a key factor contributing to the reduction in raw chili flavor. In summary, thermal treatments effectively mitigated raw chili flavor and masked the oil taste of rapeseed oil, enriching the flavor complexity of chili oil products.

## 4. Conclusions

The study employed a multi-approach integrating E-nose, GC-IMS, HS-SPME-GC-MS, LC-MS/MS, and sensory evaluation to comprehensively elucidate flavor profiles (volatile and non-volatile components) of chili powders and corresponding oils processed under three thermal treatments. Thermal treatments significantly enhanced flavor complexity and sensory acceptance by elevating Maillard reaction derivatives (e.g., ethyl pyrazine, 2-ethyl-6-methylpyrazine, 2,3-dimethylpyrazine) and modulating alcohols, aldehydes, and esters. Compared with RC, OC and MC demonstrated superior flavor enhancement effects, with these improvements persistently modulating the flavor profiles of corresponding chili oils. Notably, OCO exhibited near-universal upregulation of differential flavor compounds, indicating that prolonged low-temperature roasting effectively facilitates overall flavor release from chili. LC-MS/MS analysis further revealed heat-induced upregulation of taste-active components, contributing to improved palatability. The three thermal treatment methods not only maintained the stability of capsaicinoids, preventing a reduction in spiciness, but also significantly increased the concentration of other non-volatile flavor components. Compared to conventional raw material processing, the combination of thermal pretreatment and secondary oil-phase activation synergistically enhanced the release of flavor compounds, thereby improving both the quality and acceptability of chili products. The findings establish a scientific basis and suggest actionable pathways for improving product quality in the commercial chili processing industry. However, there are still some limitations, including a limited number of participants and a narrow age distribution, which may affect the generalizability of the results. Furthermore, the economic viability and scalability of the proposed processes, particularly regarding equipment and energy consumption, require further evaluation for industrial adoption.

## Figures and Tables

**Table 1:** Influence of different processing methods on color of chili powder and chili oil.

	L*	a*	b*	∆E
**CPs**				
RC	43.84 ± 0.17 ^a^	10.53 ± 0.36 ^b^	10.11 ± 0.53 ^b^	
SC	43.36 ± 0.65 ^a^	12.16 ± 0.63 ^a^	11.12 ± 0.77 ^ab^	2.15 ± 0.56 ^a^
OC	43.94 ± 0.64 ^a^	12.77 ± 0.57 ^a^	11.59 ± 0.67 ^a^	2.75 ± 0.80 ^a^
MC	44.14 ± 0.20 ^a^	12.96 ± 0.37 ^a^	12.09 ± 0.47 ^a^	3.15 ± 0.56 ^a^
**COs**				
RCO	26.04 ± 0.15 ^a^	12.68 ± 0.47 ^c^	4.74 ± 0.56 ^c^	
SCO	25.22 ± 0.17 ^b^	14.89 ± 0.63 ^b^	5.78 ± 0.31 ^b^	2.62 ± 0.44 ^b^
OCO	23.94 ± 0.30 ^c^	16.43 ± 0.44 ^a^	6.92 ± 0.32 ^a^	4.84 ± 0.32 ^a^
MCO	25.08 ± 0.25 ^b^	15.03 ± 0.48 ^b^	5.91 ± 0.39 ^b^	2.82 ± 0.45 ^b^

Each value was presented as the mean ± SD (n = 3) of triplicate measurements. Significance of differences in experimental outcomes was indicated by different lowercase letters within the same column (*p* < 0.05). CPs: chili powders; RC, SC, OC, and MC were untreated raw, stir-fried, oven-baked, and microwave-treated chili powder, respectively; COs: chili oils; RCO, SCO, OCO, and MCO were untreated raw, stir-fried, oven-baked, and microwave-treated chili oil, respectively.

## Data Availability

The original contributions presented in this study are included in the article/Supplementary Materials. Further inquiries can be directed to the corresponding authors.

## Supplementary Materials

The following supporting information can be downloaded at: https://www.mdpi.com/article/10.3390/foods14173129/s1, Table S1. Electronic nose sensors and its corresponding representative sensitive compounds. Table S2. The sensory evaluation criteria of different thermal treatments chili powders. Table S3. The sensory evaluation criteria of different thermal treatments chili oils. Table S4. Signal intensity of VOCs in different chili podwer samples by GC-IMS. Table S5. Signal intensity of VOCs in different chili oil samples by GC-IMS. Table S6. Identification of volatile flavor compounds of chili podwers by GC-MS. Table S7. Identification of volatile flavor compounds of chili oils by GC-MS. Table S8. Key VOCs in different chili power and oil samples detected by GC-MS. Table S9. List of the 2251 compounds (POS/NEG) in chili podwers. Table S10. List of the 1978 compounds (POS/NEG) in chili oils. Figure S1. Relative capsaicinoids content of chili powders (A), and chili oils (B). Results were presented as mean ± standard deviation (n = 3). Different letters indicate significant differences in the content of the same compound among different groups (*p* < 0.05).

## Abbreviations

The following abbreviations are used in this manuscript:

CPs	chili powders
RC	untreated raw chili powder
SC	stir-fried chili powder
OC	oven-baked chili powder
MC	microwave-treated chili powder
COs	chili oils
RCO	untreated raw chili oil
SCO	stir-fried chili oil
OCO	oven-baked chili oil
MCO	microwave-treated chili oil
E-nose	electronic nose
GC-IMS	chromatography-ion mobility spectrometr
HS-SPME-GC-MS	headspace solid-phase microextraction gas chromatography-mass spectrometry
LC-MS/MS	Liquid chromatography tandem mass spectrometry
VOCs	volatile organic compounds
