# Supplementary material for: Effects of Thermal Treatments on the Physicochemical and Flavor Profiles of Chili Powders and Their Derived Chili Oils
Source: Foods. 2025 Sep 6;14(17):3129. doi: 10.3390/foods14173129 (PMC12428499; doi:10.3390/foods14173129)
Supplement: Supplementary file 1 [file foods-14-03129-s001.zip › Supplementary Materials S1.pdf]

Supplementary Materials

Table S1

Electronic nose sensors and its corresponding representative sensitive compounds.

Sensors	Sensitive compounds
Sn_1	Propane, smoke, etc
Sn_2	Carbonaceous compounds
Sn_3	Hydrogen
Sn_4	Sulphide
Sn_5	Nitrogenous compounds
Sn_6	Aldehydes and ketones
Sn_7	Short-chain alkanes, flammable gases, etc
Sn_8	Liquefied gas
Sn_9	Alkanes, alcohols, ketones, etc
Sn_10	Hydrogen, nitrogen gas
Sn_11	Alkanes, carbon monoxide, etc
Sn_12	Liquefied gas, methane
Sn_13	Short-chain hydrocarbons
Sn_14	Methane, gas, smoke, etc
Sn_15	Carbonaceous substances, alcohols, aldehydes, etc
Sn_16	Hydrogen sulfide
Sn_17	Ammonia, amines, etc
Sn_18	Toluene, acetone, ethanol, etc

Table S2
The sensory evaluation criteria of different thermal treatments chili powders

Evaluation Criteria	Descriptive Criteria	Score (points)
Color	Vivid, pure, and uniform red color with good luster, free from any yellowing, dullness, blackening, or noticeable color unevenness	8-10
	Acceptable color, but lacking in purity (e.g., darkened, faded, slight yellow tint), with average luster, or exhibiting slight unevenness	5-7
	Dull color, severely yellowed, or blackened, or exhibiting distinct off-colors, extreme color unevenness, and lacking luster entirely	1-4
Bitterness /Off-flavor	Free from any detectable bitterness, burnt/scorched flavor, mustiness, earthy taste, metallic notes, or other unpleasant off-flavors	8-10
	Presence of slight but detectable bitterness or certain off-flavors (e.g., faint burnt note), yet not objectionable; overall flavor remains acceptable	5-7
	Pronounced and intense off-flavors (e.g., bitterness, burnt flavor, mustiness) that overwhelmingly mask the intrinsic chili character, causing distinct displeasure or even becoming intolerable	1-4
Raw Chili Flavor	Nearly imperceptible raw chili greenness or grassy notes. Exhibits rich aroma profile with well-developed characteristics (sweetness, mellow depth)	8-10
	The chili flavor is present but not intense, clear, or full-bodied. Somewhat bland or slightly vegetal/grassy, but these characteristics are not prominent	5-7
	Pronounced raw chili greenness or intense grassy flavor. Lacks processed character, imparting harsh, unripe, and distinctly unpalatable qualities	1-4
Spiciness	Moderate spiciness level with pure heat impact. Distinct upon ingestion, exhibiting appropriate persistence without scorching pain or discomfort	8-10
	Spiciness either insufficient or excessive, and/or heat character impure. Stimulus duration either too fleeting or overly prolonged	5-7
	Nearly imperceptible heat, or intolerably intense spiciness (scorching pain), or aberrant heat sensation causing significant discomfort	1-4
Overall Aroma	Rich aroma fully reflecting the characteristic scents of chili (such as baking, fruity, and floral notes), with no off-odors	8-10
	Aroma is acceptable but not rich, somewhat flat or monotonous, or with slight dissonance or off-odors that are not prominent	5-7
	Weak aroma or with noticeable unpleasant odors, such as moldy or burnt smells	1-4

Table S3

The sensory evaluation criteria of different thermal treatments chili oils

Evaluation Criteria	Descriptive Criteria	Score (points)
Color	Vibrant red oil color, clear and free from turbidity, sediment, suspended matter, or any dullness, darkening, or grayish cast	8-10
	Acceptable oil color but lacking clarity, or exhibiting slight sediment without visible suspension	5-7
	Dull, darkened, or murky oil color with noticeable sediment or suspended particles	1-4
Rapeseed Oil Flavor	Free from any raw oil, grassy, mustard, or other astringent flavors. The oil flavor is pure and free from off-odors	8-10
	Slight but perceptible raw oil, grassy, or mustard flavors are present, but not strong or off-putting	5-7
	Raw oil, grassy, and mustard flavors are very pronounced and strong, and are unpleasant	1-4
Bitterness/Off-flavor	Free from any detectable bitterness, burnt, rancid, oiliness, metallic, or raw oil flavors	8-10
	Slight but detectable bitterness, burnt, oiliness, or off-flavors are present	5-7
	Bitterness, burnt, rancid, oiliness, and other off-flavors are very pronounced and strong, severely masking the aroma of chili and oil	1-4
Spiciness	Moderate spiciness with appropriate persistence; devoid of scorching pain or discomfort.	8-10
	Suboptimal spiciness (weak/strong) accompanied by oleaginous mouthfeel and inadequate/excessive persistence	5-7
	Near-absent pungency, intolerably intense heat, or aberrant sensation causing marked discomfort	1-4
Overall Aroma	Rich and harmonious aroma, integrating the spiciness of chili and the richness of oil, with a note of baking, and no off-odors	8-10
	Aroma is acceptable but not rich or well-integrated, with slight deficiencies or separation in chili or oil notes, or minor off-odors (e.g., slight raw oil smell) that are not prominent.	5-7
	Weak aroma, with poor integration or significant separation of chili and oil notes, or noticeable off-odors (e.g., strong raw oil smell, burnt smell)	1-4

Table S4

Signal intensity of VOCs in different chili podwer samples by GC-IMS.

Compound	Formula	CAS#	M_w	RI[a]	Rt[b] [sec]	Dt[c] [a.u.]	RC	SC	OC	MC
**Aldehydes (23)**							Semi-Quantification/100			
1-nonanal-M	C9H18O	C124196	142.2	1396.4	1322.527	1.4875	34.97±3.31a	34.82±3.92a	31.82±10.41a	39.84±11.79a
1-nonanal-D	C9H18O	C124196	142.2	1395.3	1319.186	1.94567	1.90±0.17a	2.32±0.40a	2.72±1.49a	4.08±2.65a
(E)-2-Heptenal-M	C7H12O	C18829555	112.2	1327	1114.276	1.25499	23.93±3.50a	26.69±0.91a	27.66±0.61a	26.63±2.13a
(E)-2-Heptenal-D	C7H12O	C18829555	112.2	1325.1	1108.708	1.66758	9.03±2.98a	9.15±1.25a	8.56±1.41a	9.73±0.64a
(E, E)-2,4-heptadienal-M	C7H10O	C4313035	110.2	1530.6	1724.709	1.20559	70.19±5.07c	82.36±2.85b	86.44±2.59ab	91.36±5.25a
(E, E)-2,4-heptadienal-D	C7H10O	C4313035	110.2	1527.3	1714.964	1.6247	3.17±0.29a	3.10±0.26a	2.81±0.34a	2.97±0.33a
(E)-2-octenal	C8H14O	C2548870	126.2	1436	1441.215	1.33423	4.40±0.32b	6.27±0.64ab	7.27±1.70a	7.45±0.49a
(E)-2-hexen-1-al-M	C6H10O	C6728263	98.1	1219	770.944	1.17899	19.20±1.48c	20.77±0.9bc	23.06±1.29a	22.72±0.44ab
(E)-2-hexen-1-al-D	C6H10O	C6728263	98.1	1219.3	772.042	1.51293	13.02±4.11a	14.91±1.71a	18.98±3.38a	19.2±1.79a
Heptaldehyde-M	C7H14O	C111717	114.2	1186.4	670.421	1.34969	13.78±1.47a	15.87±0.65a	16.80±3.40a	18.77±4.30a
Heptaldehyde-D	C7H14O	C111717	114.2	1186.4	670.421	1.68893	3.71±0.90a	4.88±0.66a	6.26±2.95a	7.99±3.72a
(E)-2-Pentenal-M	C5H8O	C1576870	84.1	1131.5	545.064	1.11096	21.9±1.43a	19.11±1.33bc	18.62±0.72c	21.5±1.21ab
(E)-2-Pentenal-D	C5H8O	C1576870	84.1	1130.2	542.079	1.36644	38.44±6.34b	43.27±0.38b	49.55±4.37ab	58.34±7.98a
Butanal-M	C4H8O	C123728	72.1	854.6	261.933	1.11159	89.43±5.07a	88.57±2.89a	85.35±3.46a	92.72±5.79a
Butanal-D	C4H8O	C123728	72.1	859	264.428	1.28186	2.96±0.72b	4.01±0.35ab	4.67±0.85a	3.64±0.98ab
2-furaldehyde	C5H4O2	C98011	96.1	1499.6	1631.931	1.33658	17.38±2.39b	28.12±3.42ab	40.92±19.52ab	43.59±11.49a
3-Methyl-2-butenal-M	C5H8O	C107868	84.1	1203.3	719.872	1.09312	13.54±0.81ab	12.94±0.28b	14.44±1.11ab	15.25±1.34a
3-Methyl-2-butenal-D	C5H8O	C107868	84.1	1202.9	718.603	1.35636	2.61±0.33b	2.88±0.22ab	2.96±0.58ab	4.12±1.19a
n-Pentanal-M	C5H10O	C110623	86.1	982	335.165	1.17309	10.22±0.49a	9.40±0.15a	9.01±1.11a	8.67±0.81a
n-Pentanal-D	C5H10O	C110623	86.1	990.3	339.913	1.41076	71.94±9.66b	89.35±3.26a	98.59±10.01a	98.29±8.36a
(E)-2-hexen-1-al	C6H10O	C6728263	98.1	1204.6	724.337	1.18245	1.23±0.14a	1.53±0.05a	1.74±0.56a	1.67±0.24a

Table S4 (*continued*)

Compound	Formula	CAS#	M_w	RI[a]	Rt[b] [sec]	Dt[c] [a.u.]	RC	SC	OC	MC
1-pentanal	C5H10O	C110623	86.1	953.3	318.671	1.18204	45.49±2.78a	42.7±1.42a	39.72±4.75a	44.84±0.67a
(Z)-4-heptenal	C7H12O	C6728310	112.2	1216.7	763.443	1.15026	1.85±0.06c	2.21±0.25bc	2.48±0.13ab	2.61±0.21a
**Alcohols (15)**										
1 -hexanol-M	C6H14O	C111273	102.2	1361.7	1218.45	1.33075	27.33±6.87a	27.15±3.85a	25.48±2.51a	30.06±1.13a
1 -hexanol-D	C6H14O	C111273	102.2	1361.9	1218.954	1.64517	3.14±1.33a	2.99±1.00a	2.59±0.48a	3.58±0.37a
1-Pentanol-M	C5H12O	C71410	88.1	1258.4	899.365	1.25649	50.05±8.77a	57.45±1.03a	56.68±5.17a	62.6±9.09a
1-Pentanol-D	C5H12O	C71410	88.1	1258.3	898.824	1.52028	20.11±6.21a	21.81±2.77a	21.52±6.18a	29.64±10.72a
1-Butanol, 3-methyl-M	C5H12O	C123513	88.1	1208.5	736.907	1.24415	58.06±1.36a	56.45±0.65ab	54.35±1.1bc	52.48±2.15c
1-Butanol, 3-methyl-D	C5H12O	C123513	88.1	1209.6	740.656	1.49918	82.05±14.08a	87.41±3.36a	83.76±2.32a	91.45±2.3a
1- butanol	C4H10O	C71363	74.1	1141.9	568.941	1.19472	13.69±1.39a	12.88±1.30a	11.32±1.25a	12.86±0.46a
1-Propanol, 2-methyl-M	C4H10O	C78831	74.1	1095.7	464.798	1.17621	20.48±1.24a	18.81±1.02ab	17.92±1.4ab	17.35±1.33b
1-Propanol, 2-methyl-D	C4H10O	C78831	74.1	1097.1	466.683	1.38287	47.96±11.22b	55.53±1.62ab	59.19±7.5ab	66.45±7.52a
2,3-Butandiol	C4H10O2	C513859	90.1	1541	1756.074	1.35664	58.03±13.84b	98.26±4.89ab	122.62±40.2a	129.56±20.73a
(Z)-2-pentenol	C5H10O	C1576950	86.1	1322.7	1101.362	1.44902	2.15±0.31b	2.62±0.12ab	2.98±0.49ab	3.23±0.66a
3-heptanol	C7H16O	C589822	116.2	1291.2	1006.092	1.32847	78.59±25.54a	72.25±2.43a	97.64±18.52a	100.39±25.65a
2-Pentanol	C5H12O	C6032297	88.1	1106.8	488.853	1.20497	4.47±1.14a	5.03±0.69a	4.92±0.86a	5.08±0.23a
1-Penten-3-ol	C5H10O	C616251	86.1	1158.8	607.474	0.94148	36.14±1.42ab	35.07±0.95b	38.29±1.45a	36.68±0.99ab
1-Propanol	C3H8O	C71238	60.1	1036.8	392.947	1.11308	11.91±1.71a	10.75±0.66ab	8.88±1.04b	8.87±1.17b
**Esters (11)**										
Isovaleric acid, methyl ester	C6H12O2	C556241	116.2	1014.4	365.614	1.19426	7.59±0.7a	7.77±0.24a	7.89±0.25a	7.25±0.98a
Ethyl propanoate	C5H10O2	C105373	102.1	933.5	307.259	1.14624	22.53±1.33a	15.25±2.57b	12.78±3.09b	14.16±1.19b
Methyl propanoate	C4H8O2	C554121	88.1	901.9	289.084	1.08255	5.70±1.22a	5.48±0.21a	5.69±0.02a	5.94±0.50a
Butanoic acid, 3-hydroxy-, ethyl ester	C6H12O3	C5405414	132.2	1505.2	1648.52	1.15994	224.02±4.94a	227.48±2.44a	227.21±15.74a	231.1±5.36a

Table S4 (*continued*)

Compound	Formula	CAS#	M_w	RI[a]	Rt[b] [sec]	Dt[c] [a.u.]	RC	SC	OC	MC
Ethyl formate	C3H6O2	C109944	74.1	818	240.851	1.08379	48.50±0.36a	48.57±0.21a	48.35±0.37a	49.17±1.12a
1,2-Propanediol, diacetate	C7H12O4	C623847	160.2	1491.3	1606.871	1.21082	5.24±2.89a	5.36±1.22a	6.78±1.55a	6.24±0.54a
Methyl nonanoate	C10H20O2	C1731846	172.3	1534.4	1736.131	1.50514	5.62±0.61b	5.95±0.19b	7.23±0.91a	7.49±0.41a
1-Butanol, 3-methyl-, acetate	C7H14O2	C123922	130.2	1120.2	519.351	1.30333	15.38±5.03a	19.48±0.42a	18.44±2.47a	19.22±1.48a
Acetic acid butyl ester	C6H12O2	C123864	116.2	1075.8	440.515	1.2278	4.27±2.27a	5.71±0.82a	5.50±0.47a	6.79±1.31a
3-Methylbutyl 2-methylbutanoate	C10H20O2	C27625350	172.3	1294.1	1015.568	1.42269	5.56±0.34a	6.77±0.68a	5.08±0.68a	5.41±1.90a
2-Methylpropyl propionate	C7H14O2	C540421	130.2	1082.6	448.806	1.28166	10.53±0.64a	9.79±0.45a	9.13±0.6a	9.55±1.51a
**Ketone (8)**										
2-Decanone	C10H20O	C693549	156.3	1496.6	1622.935	1.47632	2.24±0.12a	2.12±0.10a	2.28±0.41a	2.37±0.14a
2-methyl-2-hepten-6-one	C8H14O	C110930	126.2	1340.7	1155.35	1.17469	8.50±1.42a	10.16±0.9ab	11.09±1.11ab	11.9±1.51b
2-Heptanone	C7H14O	C110430	114.2	1181.6	659.589	1.26356	7.17±2.99a	8.37±0.34a	7.73±0.48a	7.91±0.55a
3-Methyl-2-cyclopenten-1-one	C6H8O	C2758181	96.1	1505.7	1650.122	1.11083	182.25±7.31a	176.45±2.48a	176.37±4.56a	173.07±3.51a
2,3-Butanedione	C4H6O2	C431038	86.1	1007.2	356.862	1.17032	16.67±2.12a	16.49±1.15a	15.22±0.80a	16.66±2.51a
1-Penten-3-one-M	C5H8O	C1629589	84.1	1026.6	380.537	1.08434	11.48±0.27a	8.74±0.51b	8.22±1.39b	7.77±0.90b
1-Penten-3-one-D	C5H8O	C1629589	84.1	1024.6	378.094	1.31861	50.1±3.58ab	45.48±2.12b	48.3±2.54ab	51.6±2.71a
2-propanone	C3H6O	C67641	58.1	816.4	239.967	1.12168	7.52±0.84a	9.22±1.01a	12.16±2.22a	11.43±3.75a
**Hydrocarbons (3)**										
alpha-terpinolene	C10H16	C586629	136.2	1282.4	977.492	1.22155	0.76±0.10b	1.02±0.07a	1.13±0.13a	0.94±0.15ab
Beta-Pinene	C10H16	C127913	136.2	1113.3	503.636	1.21442	8.08±3.12a	9.36±0.85a	8.11±1.98a	7.46±2.73a
Camphene	C10H16	C79925	136.2	1045.3	403.387	1.19943	17.57±2.75b	25.32±2.10ab	28.79±7.62a	25.84±3.24ab
Gamma-Terpinene	C10H16	C99854	136.2	1248	865.282	1.212	15.37±1.26b	18.51±1.04ab	22.79±3.26a	20.6±4.78ab
**Heterocycles (8)**										

Table S4 (*continued*)

Compound	Formula	CAS#	M_w	RI[a]	Rt[b] [sec]	Dt[c] [a.u.]	RC	SC	OC	MC
2-pentyl furan	C9H14O	C3777693	138.2	1230.4	808.276	1.25229	9.34±2.94a	11.32±0.27a	11.47±1.08a	12.81±0.66a
2,3-Dimethylpyrazine	C6H8N2	C5910894	108.1	1341.8	1158.683	1.11842	4.67±0.82a	5.32±0.59a	5.07±0.81a	5.69±1.28a
2-Methylpyrazine	C5H6N2	C109080	94.1	1272.1	943.871	1.09312	5.00±0.82b	7.81±1.13a	7.66±0.82a	8.00±1.83a
2,5-Dimethylpyrazine	C6H8N2	C123320	108.1	1328.2	1117.892	1.11362	3.75±0.26b	3.91±0.19b	5.17±0.93a	6.17±0.34a
2-Ethylpyridine	C7H9N	C100710	107.2	1309.8	1062.74	1.08563	72.48±2.23b	72.77±2.41b	77.07±0.49a	74.51±0.41ab
3-Ethylpyridine	C7H9N	C536787	107.2	1376.1	1261.391	1.11524	2.90a±0.27a	3.89±0.39a	3.90±0.46a	4.08±0.96a
2H-Pyran, tetrahydro-4-methyl-2-(2-methyl-1-propenyl)-	C10H18O	C16409431	154.3	1397.1	1324.536	1.3648	57.75±27.68b	112.11±5.19ab	134.05±55.62a	140.44±28.19a
2,4,5-trimethylthiazole	C6H9NS	C13623115	127.2	1395.3	1318.971	1.14509	108.54±18.72a	132.92±1.33a	136.88±20.88a	135.27±15.94a
**Others (3)**										
(1-methylethyl) benzene	C9H12	C98828	120.2	1164.1	619.611	1.15372	15.74±0.53b	20.20±0.33a	20.12±1.31a	21.94±1.40a
Ethyl benzene	C8H10	C100414	106.2	1126.9	534.581	1.07608	9.92±0.18a	11.36±0.77a	11.76±0.24a	10.41±1.52a
Benzene, propyl-	C9H12	C103651	120.2	1193.2	687.135	1.15703	1.59±0.10c	2.23±0.11ab	2.08±0.04b	2.55±0.37a

Results were presented as mean ± standard deviation (n = 3). Different letters within the same row indicate significant differences in content among the compounds ($p < 0.05$). a: Retention indices; b: Retention times; c: Drift times.

Table S5

Signal intensity of VOCs in different chili oil samples by GC-IMS.

Compound	Formula	CAS#	M_w	RI^a	Rt^b [sec]	Dt^c [a.u.]	RCO	SCO	OCO	MCO
**Aldehydes (23)**								Semi-Quantification/100		
1-nonanal-M	C9H18O	C124196	142.2	1396.4	1322.527	1.4875	83.77±5.39a	75.90±13.27ab	60.21±1.05b	70.41±7.52ab
1-nonanal-D	C9H18O	C124196	142.2	1395.3	1319.186	1.94567	16.55±1.11a	16.84±8.03a	9.68±0.06a	13.76±4.86a
(E)-2-Heptenal-M	C7H12O	C18829555	112.2	1327	1114.276	1.25499	94.73±3.07a	89.86±1.05b	84.94±1.14c	84.03±1.21c
(E)-2-Heptenal-D	C7H12O	C18829555	112.2	1325.1	1108.708	1.66758	77.68±9.00a	80.64±2.26a	77.49±4.82a	87.45±2.01a
(E, E)-2,4-heptadienal-M	C7H10O	C4313035	110.2	1530.6	1724.709	1.20559	109.32±4.44b	115.52±2.24a	109.42±1.02b	115.26±2.82b
(E, E)-2,4-heptadienal-D	C7H10O	C4313035	110.2	1527.3	1714.964	1.6247	40.58±5.66b	46.39±2.62ab	39.74±0.51b	51.85±3.46a
(E)-2-octenal	C8H14O	C2548870	126.2	1436	1441.215	1.33423	17.34±0.55a	15.25±0.65b	13.82±0.85c	15.1±0.39b
(E)-2-hexen-1-al-M	C6H10O	C6728263	98.1	1219	770.944	1.17899	39.74±0.53a	38.89±1.05a	36.83±0.8b	38.39±0.58a
(E)-2-hexen-1-al-D	C6H10O	C6728263	98.1	1219.3	772.042	1.51293	44.99±4.63bc	51.15±2.60ab	41.70±3.82c	54.15±3.22a
Heptaldehyde-M	C7H14O	C111717	114.2	1186.4	670.421	1.34969	41.37±0.36a	40.33±1.84ab	37.73±0.80c	38.90±0.33bc
Heptaldehyde-D	C7H14O	C111717	114.2	1186.4	670.421	1.68893	29.62±2.81ab	33.34±3.63ab	28.06±2.54b	33.94±0.41a
(E)-2-Pentenal-M	C5H8O	C1576870	84.1	1131.5	545.064	1.11096	34.95±3.06a	31.76±0.39ab	30.46±0.59b	31.10±0.21b
(E)-2-Pentenal-D	C5H8O	C1576870	84.1	1130.2	542.079	1.36644	74.22±3.50ab	79.91±1.62a	68.77±6.87b	80.67±1.8a
Butanal-M	C4H8O	C123728	72.1	854.6	261.933	1.11159	72.50±6.60a	72.41±1.02a	71.78±0.72a	75.45±6.13a
Butanal-D	C4H8O	C123728	72.1	859	264.428	1.28186	8.81±1.20b	10.25±0.38ab	11.61±0.32a	9.97±1.14ab
2-furaldehyde	C5H4O2	C98011	96.1	1499.6	1631.931	1.33658	17.38±4.53b	31.96±4.73a	32.40±4.23a	23.72±3.76ab
3-Methyl-2-butenal-M	C5H8O	C107868	84.1	1203.3	719.872	1.09312	9.23±0.59b	10.89±0.26a	10.68±0.71a	11.41±0.12a
3-Methyl-2-butenal-D	C5H8O	C107868	84.1	1202.9	718.603	1.35636	2.46±0.21ab	2.88±0.15a	2.44±0.31b	2.68±0.05ab
n-Pentanal-M	C5H10O	C110623	86.1	982	335.165	1.17309	7.67±0.67b	8.04±0.09ab	8.73±0.22a	8.33±0.17ab
n-Pentanal-D	C5H10O	C110623	86.1	990.3	339.913	1.41076	113.65±5.29b	122.2±3.24b	113.78±4.28b	116.98±0.30ab
(E)-2-hexen-1-al	C6H10O	C6728263	98.1	1204.6	724.337	1.18245	5.80±0.36a	5.22±0.43ab	4.39±0.17c	4.7±0.31bc

Table S5 (*continued*)

Compound	Formula	CAS#	M_w	RI[a]	Rt[b] [sec]	Dt[c] [a.u.]	RCO	SCO	OCO	MCO
1-pentanal	C5H10O	C110623	86.1	953.3	318.671	1.18204	31.74±2.31a	34.89±0.53a	31.68±1.84a	34.06±0.74a
(Z)-4-heptenal	C7H12O	C6728310	112.2	1216.7	763.443	1.15026	10.25±0.54a	9.55±0.36b	8.33±0.16c	9.53±0.16b
**Alcohols (15)**										
1 -hexanol-M	C6H14O	C111273	102.2	1361.7	1218.45	1.33075	9.09±0.75ab	8.32±0.69b	8.18±0.52b	10.11±0.44a
1 -hexanol-D	C6H14O	C111273	102.2	1361.9	1218.954	1.64517	0.90±0.08a	0.91±0.05a	0.88±0.06a	1.02±0.10a
1-Pentanol-M	C5H12O	C71410	88.1	1258.4	899.365	1.25649	75.65±0.63a	73.13±1.74a	68.96±2.72b	73.94±0.39a
1-Pentanol-D	C5H12O	C71410	88.1	1258.3	898.824	1.52028	41.78±3.56bc	44.52±1.32b	37.23±3.37c	51.6±2.46a
1-Butanol, 3-methyl-M	C5H12O	C123513	88.1	1208.5	736.907	1.24415	18.13±0.86a	17.44±0.87a	13.15±0.22b	17.28±1.15a
1-Butanol, 3-methyl-D	C5H12O	C123513	88.1	1209.6	740.656	1.49918	5.52±0.40b	6.15±0.28ab	4.58±0.18c	6.55±0.40a
1- butanol	C4H10O	C71363	74.1	1141.9	568.941	1.19472	13.06±0.67b	13.46±0.7ab	13.19±0.35b	14.44±0.25a
1-Propanol, 2-methyl-M	C4H10O	C78831	74.1	1095.7	464.798	1.17621	7.56±0.51a	6.43±0.32b	5.89±0.03b	6.36±0.19b
1-Propanol, 2-methyl-D	C4H10O	C78831	74.1	1097.1	466.683	1.38287	8.88±1.40b	12.52±0.35a	11.86±1.20a	11.49±0.28a
2,3-Butandiol	C4H10O2	C513859	90.1	1541	1756.074	1.35664	13.25±2.36c	23.56±2.73b	25.63±2.02ab	29.64±2.40a
(Z)-2-pentenol	C5H10O	C1576950	86.1	1322.7	1101.362	1.44902	4.61±0.81a	5.28±0.21a	5.30±0.21a	5.45±0.10a
3-heptanol	C7H16O	C589822	116.2	1291.2	1006.092	1.32847	47.46±24.35a	42.06±2.40a	48.14±5.48a	58.49±15.10a
2-Pentanol	C5H12O	C6032297	88.1	1106.8	488.853	1.20497	4.97±0.70a	5.38±0.24a	5.02±0.27a	5.03±0.18a
1-Penten-3-ol	C5H10O	C616251	86.1	1158.8	607.474	0.94148	56.13±1.30a	53.74±0.23b	52.03±0.89c	52.66±0.35bc
1-Propanol	C3H8O	C71238	60.1	1036.8	392.947	1.11308	11.04±0.46a	10.93±0.03a	9.29±0.63b	11.47±0.02a
**Esters (11)**										
Isovaleric acid, methyl ester	C6H12O2	C556241	116.2	1014.4	365.614	1.19426	9.95±0.47a	10.32±0.58a	10.33±0.24a	9.9±0.24a
Ethyl propanoate	C5H10O2	C105373	102.1	933.5	307.259	1.14624	11.70±1.26a	10.54±0.82a	9.98±1.79a	10.97±0.40a
Methyl propanoate	C4H8O2	C554121	88.1	901.9	289.084	1.08255	5.63±1.58a	6.18±0.26a	5.20±0.43a	5.80±0.32a
Butanoic acid, 3-hydroxy-, ethyl ester	C6H12O3	C5405414	132.2	1505.2	1648.52	1.15994	161.27±28.11b	191.87±7.03ab	196.86±6.04a	210.82±10.8a

Table S5 (*continued*)

Compound	Formula	CAS#	M_w	RI[a]	Rt[b] [sec]	Dt[c] [a.u.]	RCO	SCO	OCO	MCO
Ethyl formate	C3H6O2	C109944	74.1	818	240.851	1.08379	45.26±2.37a	47.92±1.17a	44.96±2.67a	47.36±0.94a
1,2-Propanediol, diacetate	C7H12O4	C623847	160.2	1491.3	1606.871	1.21082	27.33±1.59a	25.03±0.56b	25.28±1.18ab	27.43±0.69a
Methyl nonanoate	C10H20O2	C1731846	172.3	1534.4	1736.131	1.50514	10.81±5.17b	19.93±0.32a	19.61±1.61a	23.01±2.19a
1-Butanol, 3-methyl-, acetate	C7H14O2	C123922	130.2	1120.2	519.351	1.30333	6.83±0.35a	6.85±0.29a	6.61±0.44a	7.22±0.16a
Acetic acid butyl ester	C6H12O2	C123864	116.2	1075.8	440.515	1.2278	2.32±0.23ab	2.54±0.04a	2.36±0.06ab	2.23±0.15b
3-Methylbutyl 2-methylbutanoate	C10H20O2	C27625350	172.3	1294.1	1015.568	1.42269	13.75±2.65a	16.44±2.92a	13.81±0.90a	15.15±2.82a
2-Methylpropyl propionate	C7H14O2	C540421	130.2	1082.6	448.806	1.28166	11.14±0.63a	9.84±0.19b	9.83±0.30b	9.69±0.10b
**Ketone (8)**										
2-Decanone	C10H20O	C693549	156.3	1496.6	1622.935	1.47632	9.57±2.29b	13.27±0.94a	13.01±1.66a	12.39±0.69ab
2-methyl-2-hepten-6-one	C8H14O	C110930	126.2	1340.7	1155.35	1.17469	4.56±0.67a	2.95±0.55b	3.96±0.20a	3.72±0.23ab
2-Heptanone	C7H14O	C110430	114.2	1181.6	659.589	1.26356	7.00±0.32ab	6.65±0.22ab	6.58±0.27b	7.21±0.28a
3-Methyl-2-cyclopenten-1-one	C6H8O	C2758181	96.1	1505.7	1650.122	1.11083	172.58±17.79a	184.97±1.02a	183.97±0.43a	181.28±1.61a
2,3-Butanedione	C4H6O2	C431038	86.1	1007.2	356.862	1.17032	6.57±0.19a	6.95±0.48a	7.18±0.27a	6.75±0.07a
1-Penten-3-one-M	C5H8O	C1629589	84.1	1026.6	380.537	1.08434	14.95±0.91a	13.22±0.33b	13.06±0.25b	13.78±0.12b
1-Penten-3-one-D	C5H8O	C1629589	84.1	1024.6	378.094	1.31861	45.45±2.24ab	49.19±1.31a	42.05±4.04b	50.06±1.36a
2-propanone	C3H6O	C67641	58.1	816.4	239.967	1.12168	2.32±0.56a	2.5±0.44a	1.82±0.33a	2.74±0.85a
**Hydrocarbons (3)**										
alpha-terpinolene	C10H16	C586629	136.2	1282.4	977.492	1.22155	4.42±2.75a	4.29±1.48a	6.07±1.97a	5.71±0.45a
Beta-Pinene	C10H16	C127913	136.2	1113.3	503.636	1.21442	3.16±0.27a	3.13±0.07a	2.88±0.16a	3.23±0.08a
Camphene	C10H16	C79925	136.2	1045.3	403.387	1.19943	57.06±3.78a	63.64±1.46a	58.08±5.29a	62.96±1.45a
Gamma -Terpinene	C10H16	C99854	136.2	1248	865.282	1.212	24.77±2.65b	29.1±1.63a	31.33±2.47a	28.95±1.26ab

Table S5 (*continued*)

Compound	Formula	CAS#	M_w	RI[a]	Rt[b] [sec]	Dt[c] [a.u.]	RCO	SCO	OCO	MCO
**Heterocycles (8)**										
2-pentyl furan	C9H14O	C3777693	138.2	1230.4	808.276	1.25229	7.58±1.16a	8.61±0.79a	7.47±0.12a	8.97±0.51a
2,3-Dimethylpyrazine	C6H8N2	C5910894	108.1	1341.8	1158.683	1.11842	7.04±0.68a	5.56±0.17b	6.21±0.53ab	5.98±0.34b
2-Methylpyrazine	C5H6N2	C109080	94.1	1272.1	943.871	1.09312	2.90±0.19b	3.83±0.21a	3.85±0.55a	3.81±0.24a
2,5-Dimethylpyrazine	C6H8N2	C123320	108.1	1328.2	1117.892	1.11362	1.59±0.28ab	1.44±0.13b	1.84±0.23ab	2.05±0.22a
2-Ethylpyridine	C7H9N	C100710	107.2	1309.8	1062.74	1.08563	82.48±6.8a	85.6±0.23a	84.00±0.94a	81.53±0.98a
3-Ethylpyridine	C7H9N	C536787	107.2	1376.1	1261.391	1.11524	3.74±0.56b	4.49±0.27ab	4.69±0.41a	4.05±0.09ab
2H-Pyran, tetrahydro-4-methyl-2-(2-methyl-1-propenyl)-	C10H18O	C16409431	154.3	1397.1	1324.536	1.3648	11.32±2.29b	20.4±1.10a	21.05±3.46a	27.43±5.62a
2,4,5-trimethylthiazole	C6H9NS	C13623115	127.2	1395.3	1318.971	1.14509	42.28±19.35b	68.2±4.01a	62.53±8.36ab	70.96±7.81a
**Others (3)**										
(1-methylethyl) benzene	C9H12	C98828	120.2	1164.1	619.611	1.15372	7.07±0.94a	6.49±0.48a	6.88±0.46a	6.75±0.31a
Ethyl benzene	C8H10	C100414	106.2	1126.9	534.581	1.07608	4.47±0.78b	4.98±0.02ab	5.48±0.15a	5.79±0.46a
Benzene, propyl-	C9H12	C103651	120.2	1193.2	687.135	1.15703	3.54±0.24ab	3.44±0.15b	3.06±0.10c	3.79±0.16a

Results were presented as mean ± standard deviation (n = 3). Different letters within the same row indicate significant differences in content among the compounds ($p < 0.05$). a: Retention indices; b: Retention times; c: Drift times.

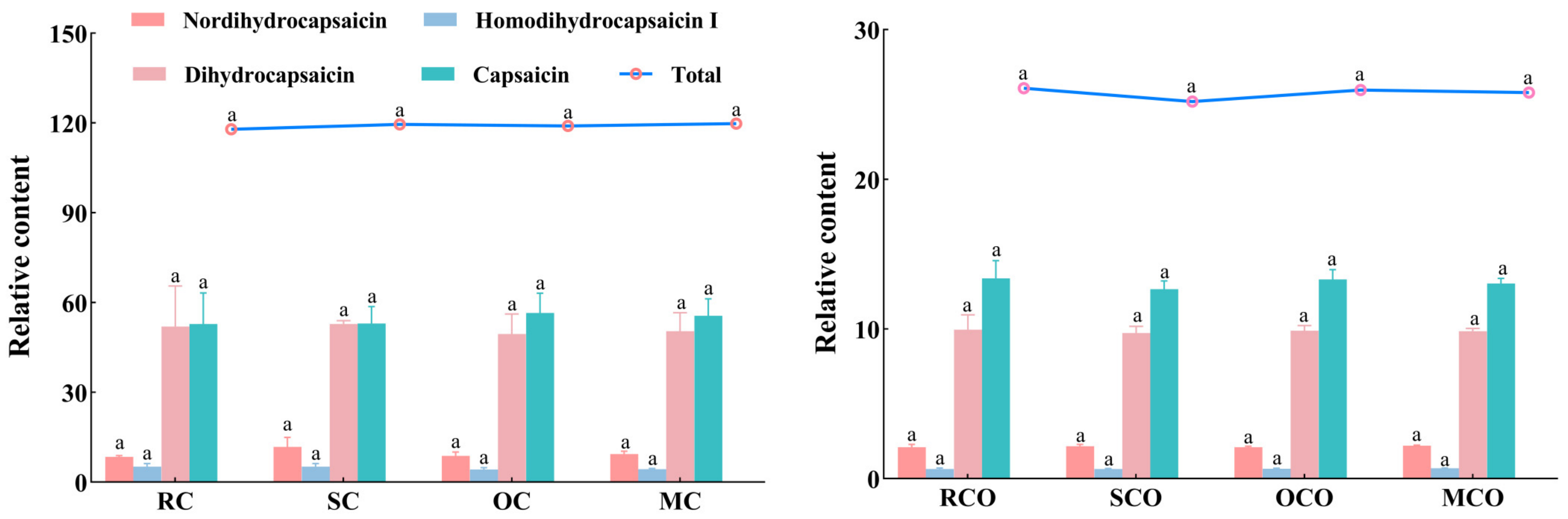

Figure S1 Relative capsaicinoids content of chili powders (A), and chili oils (B).

Results were presented as mean ± standard deviation (n = 3). Different letters indicate significant differences in the content of the same compound among different groups ($p < 0.05$).
